# The International Conference on Intelligent Biology and Medicine 2019 (ICIBM 2019): conference summary and innovations in genomics

**DOI:** 10.1186/s12864-019-6326-5

**Published:** 2019-12-30

**Authors:** Ewy Mathé, Chi Zhang, Kai Wang, Xia Ning, Yan Guo, Zhongming Zhao

**Affiliations:** 1 Department of Biomedical Informatics, The Ohio State University, Columbus, 43210 USA; 2 Department of Medical & Molecular Genetics, School of Medicine, Indiana University, Indianapolis, Indiana 46202 USA; 3 Raymond G. Perelman Center for Cellular and Molecular Therapeutics, Children’s Hospital of Philadelphia, Philadelphia, PA 19104 USA; 4 Department of Internal Medicine, Comprehensive Cancer Center, University of New Mexico, Albuquerque, NM 87131 USA; 5 Center for Precision Health, School of Biomedical Informatics, The University of Texas Health Science Center at Houston, Houston, TX 77030 USA; 6 Human Genetics Center, School of Public Health, The University of Texas Health Science Center at Houston, Houston, TX 77030 USA

## Abstract

The goal of this editorial is to summarize the 2019 International Conference on Intelligent Biology and Medicine (ICIBM 2019) conference that took place on June 9–11, 2019 in The Ohio State University, Columbus, OH, and to provide an introductory summary of the seven articles presented in this supplement issue. ICIBM 2019 hosted four keynote speakers, four eminent scholar speakers, five tutorials and workshops, twelve concurrent sessions and a poster session, totaling 23 posters, spanning state-of-the-art developments in bioinformatics, genomics, next-generation sequencing (NGS) analysis, scientific databases, cancer and medical genomics, and computational drug discovery. A total of 105 original manuscripts were submitted to ICIBM 2019, and after careful review, seven were selected for this supplement issue. These articles cover methods and applications for functional annotations of miRNA targeting, clonal evolution of bacterial cells, gene co-expression networks that describe a given phenotype, functional binding site analysis of RNA-binding proteins, normalization of genome architecture mapping data, sample predictions based on multiple NGS data types, and prediction of an individual’s genetic admixture given exonic single nucleotide polymorphisms data.

## Introduction

The 2019 International Conference on The Intelligent Biology and Medicine (ICIBM 2019), the official conference of the International Association for Intelligent Biology and Medicine (IAIBM), was co-hosted by IAIBM and the Department of Biomedical Informatics at The Ohio State University. The conference was held in June 9–11, 2019 in Columbus, OH. The goal of the conference was to foster inter-disciplinary research and discussions, educational opportunities, and collaborative efforts among the fields of bioinformatics, systems biology, and intelligent computing, and data science, which are continuing to evolve at a rapid pace and have a strong impact in scientific research and medical innovations.

Building upon the successes of previous years’ conferences [1–6], we brought together 79 researchers and 84 trainees from a wide array of national and international institutions. The backgrounds and expertise of these 163 conference participants were highly diverse, and included, among others, bioinformatics, medicine, machine learning, artificial intelligence, metabolomics, genomics, and other omics, drug repurposing, database development, and systems biology and network analysis. ICIBM 2019 thus provided a balanced mix of trainees and world-renown scientists, scientific expertise, oral and poster presentations, workshops, tutorials, and plenty of built-in breaks for invaluable discussions and networking.

A total of 105 original manuscripts and 36 abstracts were submitted. Topics covered by submissions included next-generation sequencing, single cell analyses, deep learning, metabolomics, genomics, and other omics research, systems biology, medical applications and translational research involving high-throughput data, computational methods and novel applications of computational tools, and others. Realizing the prominent impact of data science and high-throughput data in intelligent biology and medicine, ICIBM spanned current topics, with the intention of bridging topics together and fostering a cross-disciplinary environment.

Realizing that the future is in the hands of trainees, ICIBM 2019 is proud to promote participation of trainees at the conference. To this end, ICIBM 2019 received support from the National Science Foundation, through which 20 national and international trainees from various background received travel awards. Awardees were selected by an Award Committee which carefully reviewed applications and balanced the quality of the research proposed, the financial need, and the diversity of the applicants during the selection process.

In this editorial, we provide a summary of the scientific program of ICIBM 2019, and of the 7 research articles selected for the ICIBM 2019 BMC Genomics Supplement Issue.

### ICIBM 2019 scientific program

The ICIBM 2019 scientific program included 4 internationally renowned keynote speakers, 4 eminent scholar speakers, and 5 tutorials and workshops. In addition, we hosted 12 concurrent scientific sessions and a poster session with 23 accepted abstracts. ICIBM 2019 was held over 3 days, and provided multiple breaks in between sessions to foster discussions and networking.

#### Keynote speakers

ICIBM 2019 provided an exciting line-up of four world-renowned researchers that showcased their work and perspectives in advances in genome technologies, scientific databases for metabolic modeling and omics data analysis, pediatric precision cancer medicine, and human signaling networks. Speakers included 1) Dr. Jeremy Edwards, Professor in the Department of Chemistry & Chemical Biology, Albuquerque, NM, USA, 2) Dr. Peter Karp, Director, Bioinformatics Research Group, Artificial Intelligence Center, SRI, 3) Dr. Elaine Mardis, Co-executive Director of the Institute for Genomic Medicine at Nationwide Children’s Hospital and the Nationwide Foundation Endowed Chair of Genomic Medicine, Professor of Pediatrics at The Ohio State University College of Medicine, and the President of The American Association for Cancer Research (AACR) 4) Dr. T.M. Murali, Department of Computer Science, Virginia Tech, Blacksburg VA.

“*Technologies for Human Genome and Transcriptome Sequencing*” by Dr. Jeremy Edwards.

Dr. Edwards highlighted exciting technological advances that aim to sequence and assemble complex and repetitive areas of the genome. These technologies address limitations in current sequencing technologies, including limited read length and the difficulty in identifying disease-related haplotypes. His methods and tools are applied in disease-association studies where whole genome sequencing can be used to resolve haplotypes, and to more fully grasp mechanisms of the human genome and complex disease phenotypes.

“*BioCyc Tools for Metabolic Modeling and Omics Data Analysis*” by Dr. Peter Karp.

Dr. Karp showcased recent developments in BioCyc, an extensive scientific database and web portal comprising 14,500 microbial genomes and 2700 experimentally elucidated metabolic pathways from multi-organisms [7]. These developments include functionalities of the BioCyc Pathway Tools software, including the ability to develop an organism-specific quantitative metabolic model by computing a metabolic reconstruction of a defined organism from sequenced genomes. To address the issue of gaps in metabolic networks that are due to incomplete genome annotations, Dr. Karp presented a novel algorithm, for taxonomic, to fill these gaps. Further, Dr. Karp presented the newly developed Omics Dashboard, which supports integrative analysis and visualization of transcriptomic and metabolomic data.

“*A System for Pediatric Precision Cancer Medicine*” by Dr. Elaine Mardis.

Dr. Mardis highlighted her precision medicine efforts in characterizing genomic events, such as somatic alterations and germline predispositions, that lead to pediatric cancer development and progression. These ongoing efforts include the development and real-time application of DNA and RNA next-generation-sequencing (NGS)-based analysis that are used in the clinic to characterize an individual patient’s tumor type. This characterization is in turn utilized to make more accurate diagnosis, leading to personalized therapeutic decisions. Dr. Mardis highlighted specific case studies where this NGS-based strategy to precision medicine was successfully applied.

“*Pathways on Demand: Automated Reconstruction of Human Signaling Networks*” by Dr. T.M. Murali.

Dr. Murali presented his exciting work in systems biology, where he aims to make use of existing signaling pathway annotations to identify novel molecular interactions that could be included in pathways. To this end, he introduced three novel approaches, PathLinker [8], RegLinker [9], and XTalk [10], that can identify the shortest paths from receptors to transcription factors in a pathway, find the controls for non-pathway interaction in computed paths, and that produces precise networks of interactions and mechanisms, respectively. His work exemplifies that utility of automated analyses for prioritizing proteins and their interactions for further studies, and the ability of his methods to uncover novel pathways in Wnt/β-catenin signaling, which have been validated experimentally.

#### Eminent scholar speakers

“*ToppCell: A workbench for the analysis, modeling and prediction of the molecular basis of development and function of cells and tissues based on single cell atlas datasets*” by Dr. Bruce Aronow.

Dr. Aronow described novel approaches for modeling tissues as ensembles of cell types and states, to improve the ability to interpret and extract relevant biological information from single cell atlas data. To accomplish this, Dr. Aronow developed ToppCell, an open access web database that supports exploration and analysis of single cell genomic datasets. ToppCell also provides access to gene signatures from various cells, tissues, and perturbations as computable modules that can be compared against a user dataset, thereby revealing mechanisms of cell subtype functions. Modules are then modeled as networks of genes and pathways that explain functions shared by the top overexpressed genes. An adapted version of ToppCluster [11] was then utilized to identify intercellular networks, that has been shown to uncover underlying differentiation processes and new subtypes of cells in the brain and lung.

“*Identifying biologically relevant modules in metabolomics and lipidomics data with Differential Network-based Enrichment Analysis (DNEA)*” by Dr. Alla Karnovsky.

Dr. Karnovsky highlighted the Differential Network Enrichment Analysis (DNEA) method [12]. DNEA is a novel data-driven approach to constructing metabolic networks from metabolomic and lipidomic data. These networks in turn generate relevant biological insights. DNEA builds partial correlation networks using joint structural sparsity estimation, identifies highly connected subnetworks using consensus clustering, and finally uses differentially enriched subnetworks using Network-based Gene Set Analysis. DNEA has been successfully applied to public data, leading to the identification of biologically relevant interacting biomolecules that are associated with disease phenotypes.

“*The impact of sequence variants on protein function*” by Dr. Jeffrey Parvin.

Dr. Parvin presented a novel high-throughput functional analysis assay to characterize the functional impact of genomic missense substitutions. The multiplexed assay was applied to study the effect of these variants in the tumor suppressor gene BRCA1 on DNA repair function [13]. The approach yielded functional data on > 1000 missense substitutions in the amino terminus of BRCA1, which were largely consistent with the function measured by independent assays. The utility of this assay provides the ability to characterize the potential function of a large number of BRCA1 variants, including those for which the function is still unknown.

“*Prediction, searching and clustering of tandem mass spectra of peptides*” by Dr. Haixu Tang.

Dr. Tang presented his recently developed computational tools that aim to cluster, search, and predict peptide tandem mass spectra to mine large-scale MS/MS datasets [14]. These tools use machine learning to make in silico predictions of peptide MS/MS spectra directly from peptide sequences, without any prior knowledge about fragmentation rules. A variety of techniques, including run-length encoding to compress MS/MS spectra and locality sensitive hashing to index spectra, were applied to scale the algorithms and speed up the predictions. These algorithms have been shown to efficiently augment interpretation of high-throughput proteomic data.

#### Tutorials and workshops

ICIBM 2019 hosted five tutorials and workshops that covered the state-of-the-art techniques involving data science applications to cancer research, machine learning, usage of BioCyc web portal, and epigenetics data analysis.

*“Machine learning demystified*” by Dr. Yan Guo, University of New Mexico.

In this workshop, Dr. Guo introduced concepts of machine learning that are applied in biomedical research, including high throughput genomic data. Methods covered included hierarchical clustering, principal components analysis, decision trees, random forests, and finally neural networks.

“*Data driven cancer research: data science research and applications*” by Drs. Wenjin Zheng, The University of Texas Health Science Center at Houston, Yidong Chen, The University of Texas Health Science Center at Houston, and Yufei Huang, The University of Texas Health Science Center at San Antonio.

This workshop highlighted various aspects of data-driven models used in cancer research, including the analysis of a wide range of data sources from electronic records to genomic data. The workshops opened with a discussion on building an appropriate infrastructure to support such research. Live research projects used to explain different models and their use, such as clinical decision support. Three data-driven projects were also described, to exemplify how genomic data and artificial intelligence can be harnessed to discover novel cancer therapies, to predict drug combination synergies, and to predict N6-methyladenosine disease associations.

“Data driven cancer research: tutorial on deep learning for cancer genomics” by Drs. Wenjin Zheng, The University of Texas Health Science Center at Houston, Yidong Chen, The University of Texas Health Science Center at Houston, and Yufei Huang, The University of Texas Health Science Center at San Antonio.

The goal of this tutorial was to introduce the audience to the basics of deep learning, and applications of deep learning to genomics data, with a particular emphasis on cancer genomics research. This tutorial introduced the basics of deep learning and their applications, a comprehensive survey on how to generate deep learning models to analyze “omics” datasets and predict drug responses.

“*BioCyc microbial genomes web portal*” by Dr. Peter Karp, SRI International.

This tutorial showcased BioCyc, a user-friendly genome informatics portal comprising 14,700 microbial genomes. Biocyc provides information from multiple microbial databases, literature curated information, and a large suite of bioinformatics tools. The first part of the tutorial introduced BioCyc and how to interact with the web portal. The second part explained the workflow for analyzing the integration of transcriptomic and metabolomic data. Finally, Dr. Karp provided a tutorial on sing SmartTables to store, share, and analyze lists of genes and metabolites, and to perform comparative analysis across multiple organisms.

“*Machine learning for epigenomics data integration and gene regulation*” by Dr. Jianrong Wang, Michigan State University.

This workshop introduced software tools implemented a suite of machine learning algorithms. The software provides a means of integrating high-dimensional epigenomics data with transcriptomic and genomic information. Dr. Wang also introduced the mathematical basis underlying these algorithms, discussed software performance, and demonstrating it by using examples of biological applications. Four types of prediction problems were covered by these algorithms: 1) prediction of regulatory element families, 2) chromatin domain segmentation, 3) identification of targeted genes regulated by regulatory elements, and 4) prioritizing genetic variants that may cause regulation of epigenetics and genes.

#### Scientific sessions

Twelve concurrent sessions were held at ICIBM 2019, and included talks delivered by faculty, postdoctoral fellow, and PhD students. Sessions included speakers that were peer-review selected from top ranked manuscripts, spanning a wide array of expertise including bioinformatics, genomics, next-generation sequencing analysis, scientific databases, cancer and medical genomics, and computational drug discovery. We also included 2 sessions for short oral presentation to provide opportunities for high quality abstracts to present orally. Finally, one oral session was included to highlight prominent research endeavors from our international community. The twelve sessions were:

1. Next-generation Sequencing and Tools
2. General Genomics
3. Bioinformatics I
4. Next-generation Sequencing and Tools II
5. Bioinformatics II
6. Cancer Genomics I
7. Cancer Genomics II
8. Scientific Databases
9. Computational Drug Discovery
10. International Highlight Talks
11. Short Talk Session I
12. Short Talk Session II

#### Poster session

The poster session comprised 23 posters delivered by trainees and faculty. Posters represented various work highlighting bioinformatics methods and workflows leveraging molecular or clinical data, that were developed or applied to cancer biology, immunotherapy and chemotherapy, drug-drug interaction analysis, predictive modeling of prognosis or diagnosis, neurobiology, single cell analyses, interpretation and analysis of individual and integrated omics data, signaling kinetics, gene regulation of model systems (e.g. *Drosophila*), analysis of pseudogenes, and drug repurposing. Session details are available on the conference website (https://icibm2019.org/) and in the printed program book which was provided to conference participants.

#### Summaries of manuscripts in this issue

In this supplement, 7 research manuscripts were selected (out of 105 total submitted), through a review process that included at least three reviewers, followed by revisions as needed before final review and acceptance.

Li et al. [15] report a comprehensive database of potential microRNA target single nucleotide variants (SNVs) sites in the 3’UTR of mRNAs and their associated functional annotations. The goal of compiling this resource is to guide identification of putative SNVs that affect miRNA targeting, and prioritization of these sites based on their functional impact. The database is freely available at: https://sites.google.com/site/jpopgen/dbNSFP.

Ismail et al. [16] present a maximum likelihood method for reconstructing the clonal evolution of bacterial cells using time course pooled genomic sequencing data. Clonal evolution traces the accumulation of spontaneous mutations that lead to the development of novel bacterial clones. Authors evaluated their algorithms on simulated data and show that their method is superior to existing methods that do not take into account the sequential order of sequencing data.

Nguyen et al. [17] developed ManiNetCluster, a novel computational tool that aligns and clusters gene co-expression networks that are associated with specific genomic functions that are coordinated within a specific phenotype. ManiNetCluster was successfully applied to expression profiles across various model organisms as well as the time series transcriptome data. Their publicly available tool provides a novel way for uncovering the coordination of gene functions across different conditions.

Ramakrishnan et al. [18] analyzed CLIP-seq data from 60 human RNA-binding proteins (RBPs), which are critical modulators of RNA metabolism in eukaryotes. Authors demonstrate that binding sites for 1/3 of the RBPs are conserved in > 50% of the vertebrate species they evaluated. RBP binding sites were also shown to be strongly conserved across primates, and weakly conserved across birds and fishes. Authors observed that binding sites in the 3′ genic regions are more highly conserved across species than binding sites in the 5′ genic regions. Gene set enrichment analysis on highly conserved binding sites of RBPs uncovered phenotypes associated with multiple development aberrations.

Liu et al. [19] report on a new fragment length bias present in genome-wide chromatin interactions measured by genome architecture mapping (GAM) techniques. To account for this bias, authors propose normGAM, an R package that accounts for this new bias, in addition to other existing biases including window detection frequency, mappability, and GC content through the application of 5 different normalization methods. Authors compare these normalization methods and their ability to account the different biases, and observe that normalized GAM data are more strongly associated with experimental validation through fluorescence in situ hybridization experiments.

Westphal et al. [20] developed a Bayesian framework that robustly identifies matching samples based on multiple types of next generation sequencing data, including RNA-seq and MethylCap-seq. Such matching is critical, given the accidental occurrence of sample swaps that can occur in large scale omics studies. Authors demonstrate the ability of reliably identifying samples using ~ 20 million reads of RNA-seq data. They also report a reduced ability to identify samples as the sample quality and read coverage degrade.

Lastly, Wang et al. [21] tackle the issue of accurately predicting a patient’s genetic admixture by developing a computational pipeline to determine an ancestry informative marker (AIM) panel. Using ~ 1 million exonic single nucleotide polymorphisms from the 1000 Genomes Project, the authors applied an *I*_*n*_-statistic to identify the SNPs that discriminated 3 continental populations (African, European, and East Asian). They compared the discriminatory ability of their AIM panel in training and clinical datasets, and obtained superior results to those obtained by other published AIM panels.

#### Conference organization

The success of the 2019 ICIBM conference would not have been possible without the dedication of the general chair and members of the steering committee, program committee, publication committee, workshop/tutorial committee, publicity committee, award committee, trainee committee, local organization committee and industry/sponsorship committee.

Our hosts and sponsors include The Ohio State University Department of Biomedical Informatics, National Science Foundation, The University of Texas Health Science Center at Houston (UTHealth), BGI, Global Genomics Services, Macrogen, and Karger.

General Chair - Lang Li, The Ohio State University, Columbus OH, USA.

Steering Committee - Zhongming Zhao, Co-Chair, The University of Texas Health Science Center at Houston, USA; Kai Wang, Co-Chair, Children’s Hospital of Philadelphia, USA; Yidong Chen, The University of Texas Health Science Center at San Antonio, USA; Kun Huang, Indiana University; Yi Xing, Children’s Hospital of Philadelphia; Xiaohua Tony Hu, Drexel University, USA; Elmer Bernstam, The University of Texas Health Science Center at Houston.

Program Committee - Drs. Ewy Mathé, Co-Chair, The Ohio State University, Columbus OH, USA; Chi Zhang, Co-Chair, Indiana University, USA; Mehmet M. Dalkilic, Indiana University ; Sha Cao, Indiana University School of Medicine; Xia Ning, The Ohio State University; Yan Zhang, The Ohio State University; Yan Guo, University of New Mexico; Qingguo Wang, Memorial Sloan Kettering Cancer Center; Zhongming Zhao, University of Texas Health Science Center at Houston; Yan Guo, Vanderbilt University; Matthew Hayes, Xavier University of Louisiana; Lei Xie, City University of New York; Ying-Wooi Wan, Baylor College of Medicine; Shantao Li, Stanford University; Ping Zhang, The Ohio State University; Lijun Cheng, Indiana University Bloomington; Qin Ma, The Ohio State University; Yu Xue, Department of Biomedical Engineering, College of Life Science and Technology, Huazhong University of Science and Technology, Wuhan, Hubei 430074, China; Yonghui Wu, University of Florida; Sarath Janga, Indiana University - Purdue University Indianapolis; Nitish Mishra, University of Nebraska Medical Center; Xiaoli Zhang, The Ohio State University; Aimin Li, Xi'an University of Technology; Xiaoming Liu, University of South Florida; Lianbo Yu, The Ohio State University; Yin Liu, University of Texas Health Science Center at Houston; Tabrez Anwar Shamim Mohammad, Greehey Children's Cancer Research Institute (GCCRI), UTHSCSA; Lu Liu, North Dakota State University; Zhi Han, Indiana University School of Medicicne; Zhenqing Ye, Mayo Clinic; Tianbao Li, UT health of San Antonio; Ewy Mathe, The Ohio State University; Junbai Wang, Radium Hospital; Jianhua Ruan, UT at San Antonio; Hatice Gulcin Ozer, OSU Department of Biomedical Informatics; Junfeng Xia, Institute of Physical Science and Information Technology, Anhui University; Kun Huang, Indiana University School of Medicine; Zhandong Liu, Baylor College of Medicine; Lang Li, The Ohio State University; Shaojie Zhang, University of Central Florida; Han Zhang, Nankai University; Tao Li, Nankai University; Li Liao, University of Delaware; Yidong Chen, UT Health Science Center at San Antonio; Jiayin Wang, Xi'an Jiaotong University; Jingwen Yan, Indiana University - Purdue University Indianapolis; Jun Wan, Indiana University School of Medicine; Yunyun Zhou, University of Mississippi Medical Center; Guy Brock, The Ohio State University; Youping Deng, UNIVERSITY OF HAWAII; Qiwei Li, Rice University; Chaochun Wei, Shanghai Jiao Tong University; Yang Huang, Kaiser Permanente; Mirjana Maletic-Savatic, Baylor College of Medicine; Peilin Jia, UTHealth; Jun Kong, Emory University; Fuhai Li, The Methodist Hospital, Weill Medical College of Cornell University; Kai Wang, Children’s Hospital of Philadelphia; Jianlin Cheng, University of Missouri Columbia; Chi Zhang, Indiana University, School of Medicine; Zhi-Liang Ji, Xiamen University; Manabu Torii, Kaiser Permanente; Xiaofeng Song, Nanjing University of Aeronautics and Astronautics; Min Zhao, University of the sunshine coast; Yufeng Wang, University of Texas at San Antonio; Abolfazl Doostparast, University of California, Santa Barbara; Weichun Huang, NIH/NIEHS; Jim Zheng, School of Biomedical Informatics, U Texas Health Science Houston; Hua Xu, The University of Texas School of Biomedical Informatics at Houston; Yongsheng Bai, Indiana State University; Kai Wang, Children’s Hospital of Philadelphia..

Publication Committee - Yan Guo, Co-Chair, University of New Mexico; Xia Ning, Co-Chair, The Ohio State University, Columbus OH, USA.

Workshop/Tutorial Committee - Michael Wagner, Co-Chair, Cincinnati Children’s Hospital Medical Center, USA; Jin Chen, Co-Chair, University of Kentucky, USA.

Publicity Committee - Maciej Pietrzak, Co-Chair, The Ohio State University, Columbus OH, USA; Licong Cui, Co-Chair, University of Kentucky, USA.

Award Committee - Long Lu, Co-Chair, Cincinnati Children’s Hospital Center; Qiang (Shawn) Cheng, Co-Chair, University of Kentucky; Qin Ma, The Ohio State University, Columbus OH.

Trainee Committee - Tara Eicher, Co-Chair, The Ohio State University, Columbus OH, USA; Astrid Manuel, Co-Chair, The University of Texas Health Science Center at Houston, USA.

Local Organization Committee - Shasha Bai, Co-Chair, The Ohio State University, Columbus OH, USA; Lai Wei, Co-Chair, The Ohio State University, Columbus OH, USA.

Industry/Sponsorship Committee - Shiqiang Tao, Co-Chair, University of Kentucky.
