# Correction to: The International Conference on Intelligent Biology and Medicine 2019 (ICIBM 2019): conference summary and innovations in genomics

**DOI:** 10.1186/s12864-020-6753-3

**Published:** 2020-05-06

**Authors:** Ewy Mathé, Chi Zhang, Kai Wang, Xia Ning, Yan Guo, Zhongming Zhao

**Affiliations:** 1 Department of Biomedical Informatics, The Ohio State University, Columbus, 43210 USA; 2 Department of Medical & Molecular Genetics, School of Medicine, Indiana University, Indianapolis, Indiana 46202 USA; 3 Raymond G. Perelman Center for Cellular and Molecular Therapeutics, Children’s Hospital of Philadelphia, Philadelphia, PA 19104 USA; 4 Department of Internal Medicine, Comprehensive Cancer Center, University of New Mexico, Albuquerque, NM 87131 USA; 5 Center for Precision Health, School of Biomedical Informatics, The University of Texas Health Science Center at Houston, Houston, TX 77030 USA; 6 Human Genetics Center, School of Public Health, The University of Texas Health Science Center at Houston, Houston, TX 77030 USA

**Correction to: BMC Genomics 2, 1005 (2019)**

**https://doi.org/10.1186/s12864-019-6326-5**

After publication of this supplement article [1], it is requested the grant ID in the Funding section should be corrected from NSF grant IIS-7811367 to NSF grant IIS-1902617.

Therefore, the correct ‘Funding’ section in this article should read:

This article has not received sponsorship for publication. We thank the National Science Foundation (NSF grant IIS-1902617) and the Data Science and Informatics Core for Cancer Research (CPRIT grant RP170668) for the financial support of ICIBM 2019, as well as the support from Cancer Prevention and Research Institute of Texas (RP180734).
